# A Clinical Approach to Semiautomated Three-Dimensional Fetal Brain Biometry—Comparing the Strengths and Weaknesses of Two Diagnostic Tools: 5DCNS+^TM^ and SonoCNS^TM^

**DOI:** 10.3390/jcm12165334

**Published:** 2023-08-16

**Authors:** Michael Gembicki, Amrei Welp, Jann Lennard Scharf, Christoph Dracopoulos, Jan Weichert

**Affiliations:** Department of Gynecology & Obstetrics, Division of Prenatal Medicine, University Hospital of Schleswig-Holstein, Campus Luebeck, 23538 Luebeck, Germany; amrei.welp@uksh.de (A.W.); jannlennard.scharf@uksh.de (J.L.S.); christoph.dracopoulos@uksh.de (C.D.); jan.weichert@uksh.de (J.W.)

**Keywords:** fetal brain, 3D ultrasound, semiautomatic reconstruction, artificial intelligence

## Abstract

(1) Objective: We aimed to evaluate the accuracy and efficacy of AI-assisted biometric measurements of the fetal central nervous system (CNS) by comparing two semiautomatic postprocessing tools. We further aimed to discuss the additional value of semiautomatically generated sagittal and coronal planes of the CNS. (2) Methods: Three-dimensional (3D) volumes were analyzed with two semiautomatic software tools, 5DCNS+™ and SonoCNS™. The application of 5DCNS+™ results in nine planes (axial, coronal and sagittal) displayed in a single template; SonoCNS™ depicts three axial cutting sections. The tools were compared regarding automatic biometric measurement accuracy. (3) Results: A total of 129 fetuses were included for final analysis. Our data indicate that, in terms of the biometric quantification of head circumference (HC), biparietal diameter (BPD), transcerebellar diameter (TCD) and cisterna magna (CM), the accuracy of SonoCNS™ was higher with respect to the manual measurement of an experienced examiner compared to 5DCNS+™, whereas it was the other way around regarding the diameter of the posterior horn of the lateral ventricle (Vp). The inclusion of four orthogonal coronal views in 5DCNS+™ gives valuable information regarding spatial arrangements, particularly of midline structures. (4) Conclusions: Both tools were able to ease assessment of the intracranial anatomy, highlighting the additional value of automated algorithms in clinical use. SonoCNS™ showed a superior accuracy of plane reconstruction and biometry, but volume reconstruction using 5DCNS+™ provided more detailed information, which is needed for an entire neurosonogram as suggested by international guidelines.

## 1. Introduction

There is no doubt that the incremental use of artificial intelligence (AI) will transform medical imaging. In recent years, automatized software solutions applying machine learning (ML) algorithms have arrived in clinical practice. In our field, AI-driven automated image recognition and reconstruction is becoming increasingly integrated into clinical processes and is beginning to fundamentally change working and training methods in prenatal ultrasound. Automated processes can support the user in the analysis of highly complex three-dimensional anatomical structures such as the heart and central nervous system (CNS). In the field of fetal echocardiography, automatized software tools have recently been integrated into clinical examinations. Several studies indicate their potential to become firmly established as a significant part of ultrasound scans in the future [1,2,3]. It has been demonstrated that deep learning (DL) systems have good sensitivity and specificity in the diagnosis of hypoplastic left heart syndrome [3,4].

Alongside this development, similar efforts can be noticed regarding fetal neurosonography. Fetal CNS abnormalities that elude early diagnosis are among the most common reasons for referral to prenatal diagnosis [5,6]. The overall prevalence is at least 9.8 per 10,000 live births and could be higher with the use of modern imaging technologies [7,8]. The detection rates of fetal brain anomalies in an unselected population remain unsatisfactory [9] as for instance many complex CNS lesions develop throughout gestation and often become apparent later in pregnancy. Accompanied by the evolution of high-resolution ultrasound probes and the expansion of 3D ultrasound into routine diagnosis [10], the prenatal detection and diagnosis of fetal CNS anomalies have developed well, which is of great importance regarding the frequency of these entities [11]. Nevertheless, 3D ultrasound strongly depends on the expertise of the investigator and has a high intra- and interobserver variability and low reproducibility, driven by a lack of standardization [12]. The integration of automatized programs as well as the use of AI and ML algorithms in clinical routine diagnostic might help to overcome these limitations. Since 3D ultrasound programs are preinstalled on many of the commonly used ultrasound systems, they can be used for a standardized and reproducible evaluation, especially in terms of image recognition and plane reconstruction [13,14]. Additionally, several manufacturers provide onboard software tools (e.g., SonoCNS™, Smart Planes CNS™, 5DCNS+™), which allow a standardized semiautomatic plane reconstruction and biometric measurement of the fetal CNS. Recent studies were able to show a principal feasibility of those software tools using AI algorithms for prenatal biometric measurement [9,13,15,16,17].

The aim of this study was to assess and compare the feasibility of biometric measurements in structurally normal second- and third-trimester fetuses using two postprocessing tools, 5DCNS+™ and SonoCNS™. In addition, the particular strengths and weaknesses of the two programs were examined.

## 2. Materials and Methods

### 2.1. Study Population

The volumes used for this prospective study were acquired from women undergoing routine second- and third-trimester ultrasound at the department of prenatal ultrasound at the University Hospital of Schleswig-Holstein, Campus Luebeck, between April 2020 and May 2021.

The study included 133 women, each of whom was scanned by an expert in fetal sonography (J.W.) with two ultrasound machines to obtain 3D volumes using both the SonoCNS™ (Version BT20EC350) and 5DCNS+™ (Version 1.03.01.3506) programs. In addition, biometric measurements were performed conventionally with one of the two ultrasound machines. All the examinations were performed transabdominally during a routine targeted anatomical survey using a Samsung HERA W10 ultrasound system (Samsung Medison, Seoul, Republic of Korea) equipped with a 1–8 MHz curved transducer (CV1-8A) and a GE Voluson E10 ultrasound system (GE Healthcare, Chicago, IL, USA) equipped with a 2–6 MHz curved transducer (RM6C-D). We included structurally normal second- and third-trimester singleton fetuses undergoing detailed anatomy ultrasound scan. The study was approved by the local ethics committee and informed consent by all the patients was given.

### 2.2. SonoCNS™

**Figure 1:**
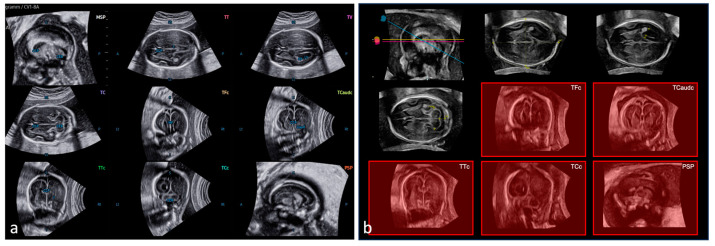
Diagnostic multi-image template of the fetal CNS anatomy at 20 + 3 weeks generated by 5DCNS+™ (panel (**a**)) and SonoCNS™ (panel (**b**)). The red-shaded planes (coronal and parasagittal) are generated manually using OmniView™ and are not reconstructed automatically by SonoCNS™.

Using SonoCNS™, the examiner needs to depict the transthalamic plane as needed for correct conventional quantification of the biparietal diameter (BPD), switch to 3D mode and apply SonoCNS™. The acquisition angle ranged from 70 to 85° and the highest-quality setting was used. In the case of fetal or maternal movements, the acquisition process was interrupted and restarted. After successful volume acquisition, the software immediately reconstructs three axial (transverse) planes (i.e., transthalamic, transventricular and transcerebellar planes, respectively), corresponding to a basic CNS examination. It further provides automatic measurement of head circumference (HC), frontooccipital diameter (FOD), BPD, posterior horn width of the lateral ventricle (Vp), transcerebellar diameter (TCD) and width of cisterna magna (CM). If necessary, the reconstructed planes can be adjusted manually, with the same applying to the biometric measurements. The planes reconstructed by SonoCNS™ are shown in Figure 1. The process of generating the planes using SonoCNS™ is shown in the Supplementary Materials.

### 2.3. 5DCNS+™

Application of the semiautomatic tool 5DCNS+™ following 3D volume acquisition as described above with the fetal skull orientated in the transthalamic plane resulted in a triplanar orthogonal reconstruction of the fetal central nervous structures. After manual marking of both the thalamic nuclei (1st seed) and cavum septum pellucidi (2nd seed) in an axial plane, a nine-image template is automatically retrieved, as shown in Figure 1. In addition to all the axial views, the software enables the assessment of the coronal and sagittal diagnostic planes. In the axial planes, HC, FOD, BPD, Vp, TCD and CM are measured automatically. If necessary, the reconstructed planes and biometric measurements can be adjusted manually, as reported elsewhere [13]. The process of generating diagnostic planes using 5DCNS+™ is shown in the Supplementary Materials.

The abbreviations used are as follows: (MSP) midsagittal plane, (TT) transthalamic plane, (TV) transventricular plane, (TC) transcerebellar plane, (TFc) transfrontal plane (coronal), (TCaudc) transcaudate plane (coronal), (TTc) transthalamic plane (coronal), (TCc) transcerebellar plane (coronal), (PSP) parasagittal plane, (CSP) cavum septi pellucidi, (CER) cerebellum, (T) thalamus, (CP) choroid plexus, (LV) lateral ventricle, (IHF) interhemispheric fissure, (Caud) caudate nucleus, (HC) head circumference, (BPD) biparietal diameter, (CM) cisterna magna, (A) anterior, (P) posterior, (S) superior, (I) inferior, (Lt) left and (Rt) right.

### 2.4. Analysis by the Operator

All the volumes were analyzed using the two software tools as previously described, and the automatic biometric measurements were compared to the manually determined values, which were rated as the gold standard. In addition, the need for manual adjustment of the planes was registered and applied, if necessary (J.W.). The measurements generated by the software tools were not adjusted. The included volumes and created planes had to match certain quality requirements (e.g., minimal or absent shadowing, a clearly visible cerebellum, absent fetal movement and adequate image clarity). The quality was judged by one expert investigator (J.W.).

### 2.5. Statistics

Data were investigated regarding the differences between the manual measurements and the measurements taken by the programs. GraphPad Prism 9 for Mac (Version 9.5.0, GraphPad Software Inc., La Jolla, CA, USA), and Microsoft Excel for Mac (Version 16.69.1, Microsoft Corp., Redmond, WA, USA) were used. Descriptive statistics were applied, and data were compared using Bland–Altman plots. A Bland–Altman plot is a scatter plot of the difference between the two measurements (Y-axis) against the average of the two measurements (X-axis). It provides a graphical display of bias (mean difference between the two techniques) with 95% limits of agreement (limits of agreement = mean observed difference ± 1.96 × standard deviation of observed differences). If there is good agreement between the measurements, then the plot should have the following features: mean difference close to zero, differences randomly scattered around zero, with no obvious pattern or trend and limits of agreement should be within clinically unimportant boundaries.

## 3. Results

A total of 129 cases were enrolled for final analysis, after excluding four cases with cerebral anomalies. The mean gestational age was 22.9 weeks (ranging from 17.6 to 35.9 weeks), the mean maternal age was 32.7 years (ranging from 19 to 44 years) and the mean body mass index was 27.2 kg/m^2^ (ranging from 19.5 to 46.1 kg/m^2^). The average number of attempts needed to acquire a sufficient volume data set was 1.14 for 5DCNS+™ and 1.05 for SonoCNS™, respectively. Most of the planes could be reconstructed satisfactorily with the respective software tool, even though the need for manual adjustment of the axial planes was higher using 5DCNS+™ (20.9 vs. 3.1%). The dropout rates (no adequate plane reconstruction after manual adjustment) were 0.75 and 0.9%, respectively (comparing 5DCNS+™ to SonoCNS™).

**Figure 2:**
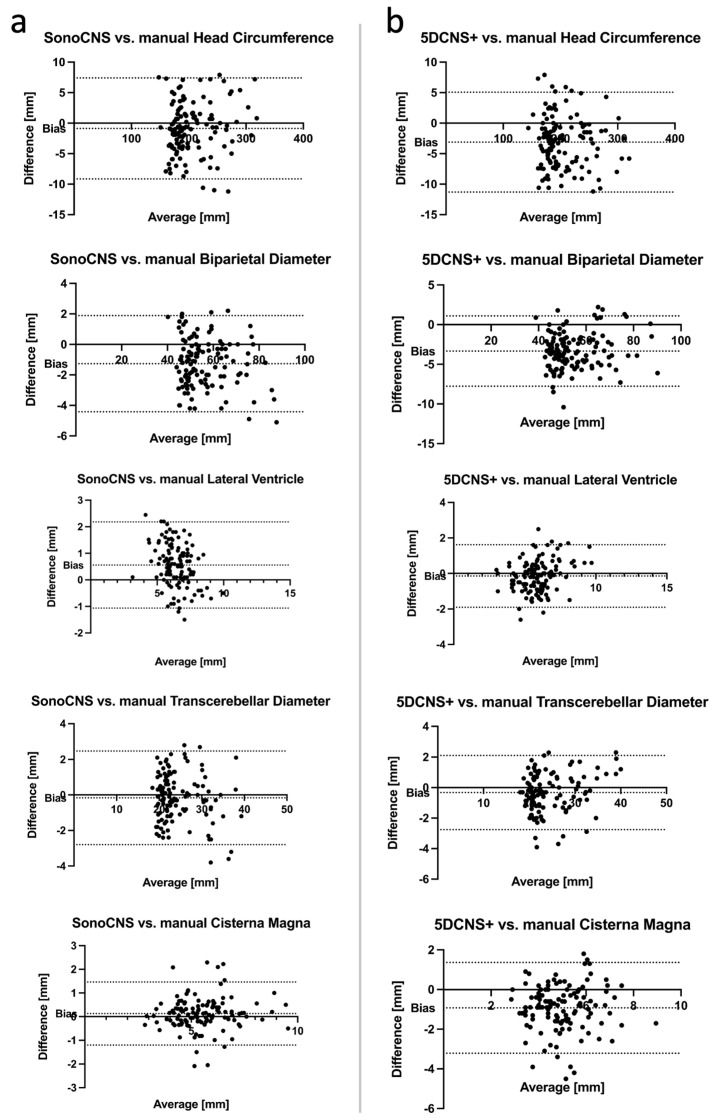
Bland–Altman plots of the differences between the manual measurements and the measurements taken by the programs SonoCNS™ (**a**) and 5DCNS+™ (**b**) for head circumference, biparietal diameter, posterior horn of the lateral ventricle, transcerebellar diameter and cisterna magna. Dotted lines represent bias (mean difference between the two techniques) and 95% limits of agreement (limits of agreement = mean observed difference ± 1.96 × standard deviation of observed differences). Note that SonoCNS™ showed lower deviation (a smaller bias) than 5DCNS+™ for head circumference, biparietal diameter, transcerebellar diameter and cisterna magna. In contrast, 5DCNS+™ showed a lower deviation for the lateral ventricle.

To compare the biometric measurements (5DCNS+™ vs. manual and SonoCNS™ vs. manual), Bland–Altman plots were created (see Figure 2). The analysis of the Bland–Altman plots indicated a higher accuracy of SonoCNS™ in terms of the biometric quantification of HC (bias −0.8713 mm ± SD of bias 4.222 mm vs. −3.117 ± 4.176 mm), BPD (−1.265 ± 1.609 mm vs. −3.339 ± 2.260 mm), TCD (−0.1623 ± 1.344 mm vs. −0.3262 ± 1.240 mm) and CM (0.1310 ± 0.6796 mm vs. −0.9261 ± 1.168 mm) in comparison to 5DCNS+™. In contrast, SonoCNS™ showed a lower accuracy in biometric quantification of Vp (0.5577 ± 0.8279 mm vs. −0.1439 ± 0.8981). All the values can be seen in Table 1. In addition, SonoCNS™ showed slightly more values within 95% limits of agreement compared to 5DCNS+™. Both tools had differences with the manual measurement randomly scattered around zero, with no obvious pattern.

**Figure 3:**
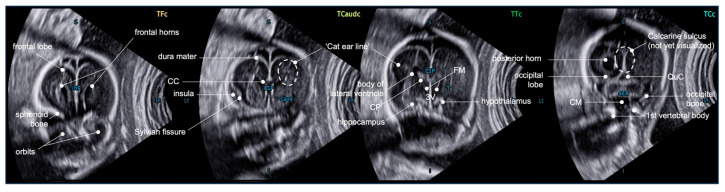
Coronal planes of the fetal CNS automatically derived from 3D volume data at 20 + 3 weeks following application of 5DCNS+™. Note that the anatomical structures labeled in white have been manually marked for illustrative purposes showing the additional diagnostic value of the coronal planes.

In the four coronal planes (transfrontal, transcaudate, transthalamic and transcerebellar, see Figure 3) additionally provided by 5DCNS+™, cerebral structures (e.g., thalamus, lateral and third ventricle and basal ganglia as well as posterior fossa structures) could be identified clearly. Midline structures, e.g., corpus callosum or cingulate cortex, could be evaluated in the sagittal planes.

The following abbreviations are used: (TFc) transfrontal plane (coronal), (TCaudc) transcaudate plane (coronal), (TTc) transthalamic plane (coronal), (TCc) transcerebellar plane (coronal), (IHF) interhemispheric fissure, (CSP) cavum septi pellucidi, (Caud) caudate nucleus, (T) thalamus, (CER) cerebellum, (CC) corpus callosum, (CP) choroid plexus, (FM) foramina of Monro, (3V) third ventricle, (CM) cisterna magna, (QuC) quadrigeminal cistern (S) superior, (I) inferior, (Lt) left, (Rt) right. For ‘Cat ear line’ see ref. [18].

## 4. Discussion

### 4.1. Biometric Measurement of Fetal Brain

The primary aim of this study was the evaluation of two automated ultrasound algorithms in a clinical setting enabling the operator to perform a standardized and reproducible examination of the fetal brain anatomy including fetal CNS biometry. Our data indicated the applicability of two different volumetric approaches for an efficient and reproducible biometric measurement of important fetal CNS structures. Compared to a manual measurement, SonoCNS™ showed a smaller deviation than 5DCNS+™ for HC, BPD, TCD and CM. In contrast, 5DCNS+™ showed better results for Vp. All the measurements quantified by both algorithms showed some underestimation compared to the manual measurements, except Vp and CM, whose values were slightly overestimated by SonoCNS™. In terms of clinical significance, all the Bland–Altman plots showed mean differences close to zero and the differences were randomly scattered around zero, so that no obvious pattern could be observed. The limits of agreement were rated to be within clinically non-critical boundaries.

### 4.2. Importance of Accurate Biometry

The importance and challenges of accurate fetal biometry have been extensively discussed and investigated in the past. However, reliable values for estimated birth weights are difficult to obtain. The number and equation variations of different fetal weight assessment models are extensive. For example, the clinical performance of 26 different sonographic birth weight prediction models was examined by Melamed et al. and showed high variation within 3 days of delivery [19]. Birth weight constitutes a predictive parameter for neonatal morbidity and mortality [20]. There is a strong association between birth weight and a wide range of health outcomes, e.g., the mortality risk during the first year after delivery [21,22]. Particularly in cases of fetal macrosomia, a condition that is often related to an increased risk of neonatal complications [23], an exact measurement is important [24,25]. Our study showed that the automatically determined biometric measurements had a minimal difference compared to the manual measurements and the limits of agreement were within clinically unimportant boundaries, so that accurate and precise measurements were created by both software tools, with slightly higher accuracy for SonoCNS™. This, in turn, justifies the implementation of both software tools into a clinical work-up as reliable, timesaving and cost-effective solutions. Accordingly, recent publications have suggested that automated biometric measurements in general might help to reduce interobserver variability by increasing the standardization of crucial measurements. Pluym et al. demonstrated a sufficient accuracy of HC and BPD measurement by applying an automated 3D ultrasound technique (SonoCNS™) whereas the measurement of Vp, TCD and CM still required improvement [9]. Other approaches focused on measuring and investigating the fractional limb volume to improve the accuracy and precision of fetal birth weight estimation [26,27,28], showing inconsistent results.

An exact qualitative and quantitative evaluation of the lateral ventricles (e.g., overall appearance, echogenicity of the ventricular wall and width of the posterior horn (Vp)) is recommended in national and international guidelines [29,30,31,32] to rule out anomalies like an enlargement of the ventricles (ventriculomegaly), a common hint for underlying CNS malformations [33]. Asymmetric ventricles as well as abnormal cerebellar shape and size and general arrangement of the posterior fossa may also refer to additional CNS abnormalities and necessitate a detailed neurosonogram [34]. The automatic reconstruction of the transcerebellar plane readily enables both the accurate measurement of the fetal cerebellum and the assessment of the entire posterior fossa including the dimensions of the cisterna magna. Thus, the majority of fossa posterior anomalies, e.g., vermis hypoplasia or mega cisterna magna, can be ruled out in the case of a normal cerebellar anatomy [35,36]. In the event of late presentation for screening or unknown last menstruation, this measure can be used as a proxy to determine the gestational age [37].

### 4.3. Additional Value of the Coronal Planes

As mentioned above, in addition to biometric assessment, the spatial arrangement and qualitative assessment of most anatomical CNS structures like the cerebellum, the thalami and the ventricle system could be readily evaluated applying both software tools. In the transcerebellar plane, a profound evaluation of the cerebellum und cisterna magna can be performed including the visualization of the cerebellar vermis. Given a high image resolution, even subtle anomalies could potentially be detected. Nevertheless, by including both coronal and sagittal planes in the diagnostic work-up, there is an increased probability of delineating abnormal CNS anatomy as provided by 5DCNS+™ only, corroborating the additional value of orthogonal diagnostic views. To take full advantage of an automated approach based on 3D volume data sets, we believe that the implementation of additional (coronal) planes is essential, as clearly recommended by the revised ISUOG practice guidelines for the sonographic examination of the fetal central nervous system [31,32].

Midline structures like the thalami, the (peri-)callosal region, the gyrus cinguli and the third and fourth ventricles can be visualized especially well in the sagittal planes, whereas a profound assessment of the cerebellum, the basal ganglia and the third ventricle is possible in the coronal planes [13,15], as illustrated in Figure 3. This underpins the importance of a multiplanar assessment of the fetal brain based on 3D ultrasound, since the evaluation of midline structures and the integrity of the anterior and posterior complex might be hampered in the case of an unfavorable fetal position [38]. It is important to notice that with 5DCNS+™ the need for manual adjustment of the reconstructed planes was higher in these planes compared to the axial planes (30.2 vs. 20.9%), headed by the parasagittal plane. In our eyes, there is a certain diagnostic value given by these planes but improvement in terms of more accurate automated plane reconstruction is necessary.

In addition, subtle changes in the gyration and sulcation process [39,40,41] can be detected in these planes during repeated follow-up scans throughout advancing pregnancy, particularly from week 20 onward. Here, the characteristic development of the lateral sulcus, calcarine sulcus and insula, respectively, is clearly comprehensible. Poon et al. argued that it is rather difficult to develop a sonographic screening tool based on a transvaginal approach, which is limited exclusively to fetuses with cephalic presentation [41]. A standardized transabdominal assessment of the fetal Sylvian fissure and its opercularization as described by Quarello et al. [42] is feasible using both software tools and might be incorporated into routine screening programs. The resulting additional diagnostic value needs to be emphasized clearly, as these regions might evade the sonographer’s examination, especially in the case of limited neurosonographic expertise.

### 4.4. Image Quality

As mentioned above, the volumes and planes had to match certain quality requirements (e.g., minimal, or absent shadowing, a clearly visible cerebellum, absent fetal movement, and adequate image clarity). The image quality was higher in 5DCNS+™. Interestingly, the initial transthalamic plane to start the reconstruction by the algorithms needs to be adjusted more accurately for SonoCNS™ to sufficiently generate the diagnostic planes.

### 4.5. Similar Approaches

A different approach to volume-based neurosonography is the Virtual Organ Computer-aided AnaLysis (VOCAL), which is typically used to calculate the volume of cystic structures. A recent study used VOCAL to evaluate intra- and interobserver agreement for the measurement of intracranial, cerebellar and thalamic volume, showing inconsistent results [43]. The immediate added diagnostic value of VOCAL in fetal neurosonography must be questioned, as it does not allow clinically necessary biometric measurements to be performed, much less diagnostic planes to be displayed. Another technical solution addresses the problem that in axial planes the hemisphere facing the transducer might be partially obscured by shadows. Especially in the late stages of pregnancy with increasing calcification of the fetal scull, this might hamper sufficient evaluation of the fetal brain in this area. Perez-Gonzalez et al. developed a method for shadow suppression by using ‘probabilistic spatial compounding’ [44], a technique that combines ultrasound images obtained from different directions into a single compound image.

Besides the programs we have investigated, there are only a few volume-based automated software tools that are commercially available and thus accessible to the general public. A similar approach is used by Smart Planes CNS™, commercially available software that offers AI-based assistance for plane reconstruction and fetal brain biometry. It was recently evaluated, and it was concluded that it would currently be a potential adjunct to the manual approach, but not a replacement for it [45]. Other authors described Smart Planes CNS™ as a reliable tool with correct plane depiction and precise biometric measurements [16]. However, similarly to SonoCNS™, it also provides only four diagnostic planes (three axial and one midsagittal) [45].

Volumetric approaches might have the potential to be used on a large scale because 3D ultrasound is reported to be a cheap, simple and accessible technique [46]. The stored volumes can be used for teaching, training and remote solutions [13,14,47]. In fact, assisting tools like SonoCNS™ and 5DCNS+™ might effectively aid in detailed neurosonographic examination, which is of great importance, since a broad variability in quality and evaluability of ultrasound scans has been reported [48]. It is of note that SonoCNS™ provides only four axial planes and can be used for screening during a basic examination, while a full neurosonogram as recommended by the ISOUG Guidelines [31,32] can be performed with 5DCNS+™ only.

Currently published promising research even went beyond advanced semiautomatic algorithms, using the DL technique for automatic fetal cerebellum segmentation from 2D ultrasound images and MRI imaging studies to replace the existing time-consuming and inaccurate measures [49,50]. A recent study demonstrated the feasibility of subcortical segmentation in 3D CNS ultrasound volumes using DL and showed that the volumetric measures obtained from these models could be used to improve understanding of subcortical growth during gestation. Furthermore, research is going on to evaluate the feasibility of ML tools for segmenting and classifying first-trimester fetal brain ultrasound images to lay the foundations for the earlier diagnosis of fetal brain abnormalities [51].

### 4.6. Strengths and Limitations

Our study has strengths and limitations. All the examinations were performed prospectively at one single tertial center by one investigator with high expertise in fetal neurosonography. Therefore, the consistency of the data is high. In contrast, less-experienced operators might obtain a higher variability of biometric measurements and create a need for manual adjustment. We included high-quality ultrasound volumes and pictures. However, a high-quality volume depends on a good acquisition plane and, to achieve this, training and experience are required. In addition, post-processing, recognition and the assessment of structures are possible only if the examiner has knowledge of physiological and pathological anatomy. The inclusion of obese patients can be considered as a strength since this condition reflects our cross-sectional population well and might make our findings more generalizable. Another limitation is the relatively small sample size. Still, the elaborated study design with two separate scans on two machines during a single appointment to generate paired observations is of high value. Our study had a certain selection bias because not all possible patients within the study period were scanned with 3D ultrasound for various reasons. The patients were enrolled into our study only when the investigator and the ultrasound machines were available at that moment. Nevertheless, our study population reflects quite well the patients we are taking care of in daily practice.

## 5. Conclusions

Summarizing, both programs are valid and efficient tools to enable the operator to perform a profound evaluation of fetal brain. A basic examination and biometry of the fetal brain can be obtained within seconds with satisfying results with advantages for SonoCNS™. A full neurosonogram as suggested by current guidelines can be achieved automatically only by 5DCNS+™. In our eyes, AI-powered software solutions can help both the inexperienced and the experienced examiner. The inexperienced examiner can be supported in terms of a more accurate basic examination and biometry, and the experienced examiner can be supported in performing a full neurosonogram to save limited resources such as time and costs. However, advanced software solutions are not yet generally accessible and implemented in routine clinical practice, so conventional neurosonography must continue to be taught and learned. Especially when considering such programs to work as a screening tool, the complete workflow from acquiring the volumes to postprocessing them must be reliable in non-expert as well as in expert hands. The ongoing development in this field will improve software-based solutions rapidly and further studies are necessary to establish their clinical usefulness. For example, it may be of interest to examine the time spans required for a complete CNS assessment using AI by experienced neurosonographers as well as by average obstetric sonographers, or to examine the time required for a complete CNS assessment by experienced neurosonographers with and without the use of AI.

## Figures and Tables

**Table 1:** Bias and standard deviation (SD) of bias generated by the Bland–Altman plots of the differences between the manual measurements and the measurements taken by the programs SonoCNS™ and 5DCNS+™ (head circumference (HC), biparietal diameter (BPD), posterior horn of the lateral ventricle (Vp), transcerebellar diameter (TCD), cisterna magna (CM)).

	SonoCNS™		5DCNS+™	
	Bias [mm]	SD of Bias [mm]	Bias [mm]	SD of Bias [mm]
HC	−0.8713	4.222	−3.117	4.176
BPD	−1.265	1.609	−3.339	2.260
Vp	0.5577	0.8279	−0.1439	0.8981
TCD	−0.1623	1.344	−0.3262	1.240
CM	0.1310	0.6796	−0.9261	1.168

## Data Availability

The data presented in this study are available on request from the corresponding author. The data are not publicly available due to data privacy restrictions.

## Supplementary Materials

The following supporting information can be downloaded at: https://www.mdpi.com/article/10.3390/jcm12165334/s1. SonoCNS™ screen video: Illustrating the process of generating diagnostic planes of the fetal CNS at 26 + 4 weeks by using SonoCNS™. The examiner depicts the axial transthalamic plane in conventional two-dimensional (2D) ultrasound, switches to 3D mode and applies SonoCNS™. The algorithm automatically acquires a 3D volume and immediately reconstructs the midsagittal and three axial planes (transthalamic, transventricular and transcerebellar plane) corresponding to a basic CNS examination. The program further provides automatic measurement of head circumference (HC), frontooccipital diameter (FOD), biparietal diameter (BPD), posterior horn width of the lateral ventricle (Vp), transcerebellar diameter (TCD) and width of cisterna magna (CM). The examiner then manually adjusts the HC. Manual adjustment can be performed for any plane or biometric measurement, if necessary. Finally, the examiner switches to the triplanar orthogonal view and manually depicts the coronal transcaudate plane. 5DCNS+™ screen video: Illustrating the process of generating diagnostic planes of the fetal CNS at 20 + 3 weeks by using 5DCNS+™. The examiner applies 5DCNS+™ following 3D volume acquisition with the fetal skull orientated in the transthalamic plane resulting in a triplanar orthogonal reconstruction of the fetal central nervous structures. After manual adjustment and marking of both the thalamic nuclei (1st seed) and cavum septum pellucidi (2nd seed) in an axial plane, a nine-image template with all diagnostic planes of the fetal CNS is automatically retrieved. Note that the marking process is supported by sample drawings displayed next to the actual image. In the axial planes, head circumference (HC), frontooccipital diameter (FOD), biparietal diameter (BPD), posterior horn width of the lateral ventricle (Vp), transcerebellar diameter (TCD) and width of cisterna magna (CM) are measured automatically. If necessary, the reconstructed planes and biometric measurements can be adjusted manually. In the video, this was performed for the parasagittal plane.
